# Bronchobiliary fistula caused after percutaneous transhepatic biliary drainage treatment: A case report

**DOI:** 10.1097/MD.0000000000036363

**Published:** 2023-12-15

**Authors:** Bo-Da Lian, Wen-Yi Zhou, Jiang Peng, Xin Zhang, Kang Zhao, Chen Chen, Xin-Tian Wang, Yong-Gang Wang, Zi-Li He

**Affiliations:** a Department of Hepatobiliary Surgery, Hunan Provincial People's Hospital (The First Affiliated Hospital of Hunan Normal University), Changsha, Hunan, China; b Department of Ultrasound Interventional, Hunan Provincial People's Hospital (The First Affiliated Hospital of Hunan Normal University), Changsha, Hunan, China.

**Keywords:** bronchobiliary fistula, case report, drainage, percutaneous transhepatic biliary, therapeutic

## Abstract

**Rationale:**

Percutaneous transhepatic biliary drainage (PTBD) plays a significant role especially in the diagnosis and decompression of bile duct obstruction. However, it is associated with complications such as hemobilia, occlusion of drainage, bile leakage, and even bronchobiliary fistula (BBF).

**Patient concerns and diagnoses:**

We herein describe a patient with a complication of BBF caused by long-term indwelling PTBD catheters. She underwent multiple operations including bilioenteric anastomosis, hepatic left lateral lobectomy, and long-term PTBD treatment. Her symptoms were mainly cough, fever, and yellow sputum and her diagnosis was confirmed by sputum culture (bilirubin detection was positive).

**Interventions and outcomes:**

The patient recovered uneventfully by minimally invasive treatment, was discharged after 1 week of hospitalization, and the drainage tube was removed 2 weeks later. During 2 years of follow-up, no recurrence of BBF was observed.

**Lessons:**

Patients with long-term indwelling PTBD catheters for biliary tract obstruction may lead to BBF. The treatment plan of BBF is tailored to the patient’s individualized characteristics. And minimally invasive treatments might be an effective alternate way for the treatment of BBF. The accurate diagnosis, precision treatment, and multidisciplinary team play important roles in the treatment of BBF.

## 1. Introduction

Bronchobiliary fistula (BBF) is a rare complication of infection, trauma, and complex surgical procedures,^[1–3]^ which is defined as an abnormal intercommunication between the biliary tract and the bronchial tree,^[4]^ with high mortality and morbidity rate (12.2%).^[5,6]^ Multiple management strategies have been described in the literature including complex surgical procedures, minimally invasive techniques, and systemic treatment.^[7–9]^ In this article, a medical record of a patient with BBF successfully recovered through minimally invasive treatment is reported.

## 2. Case presentation

### 2.1. Medical history and physical examination

A 55-year-old woman was admitted to our hospital with a 15-day history of fever (the peak temperature of 39 °C), yellow sputum (50–100 mL per day), chest tightness, and persistent cough.

She had an extensive surgical history (bilioenteric anastomosis, hepatic left lateral lobectomy), and undergone a long-term percutaneous cholangiocentesis procedure during 6 months. Physical examination revealed localized tenderness in the right upper abdomen. Lung auscultation showed moist crackles at the base of the left lung.

### 2.2. Examination and diagnosis

**Figure 1.**
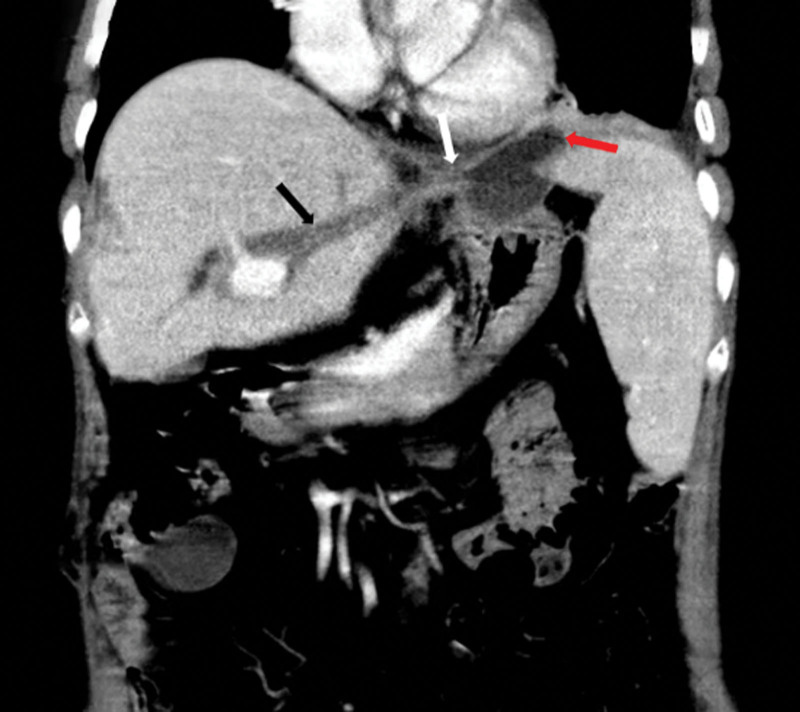
CT image scans revealed intrahepatic bile duct calculi, and biliary tract dilation (Black arrow); dilated bile duct is connected to the subphrenic abscess (White arrow); and the subphrenic abscess contained gas (red arrow). CT = computed tomography.

**Figure 2.**
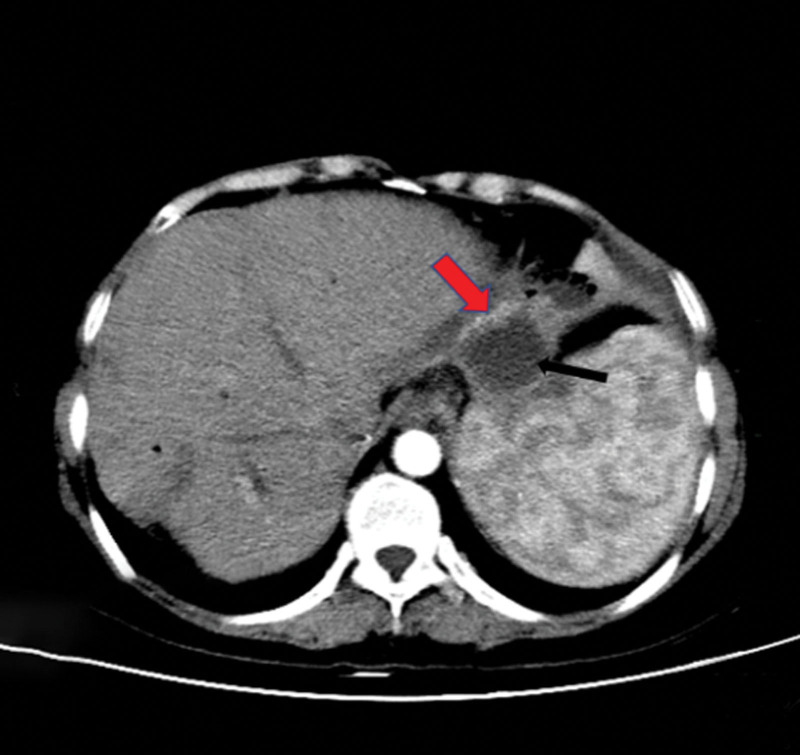
Enhanced CT image scans demonstrate a subphrenic abscess (Black arrow); the inferior phrenic artery passes from the front of the subphrenic abscess (red arrow). CT = computed tomography.

**Figure 3.**
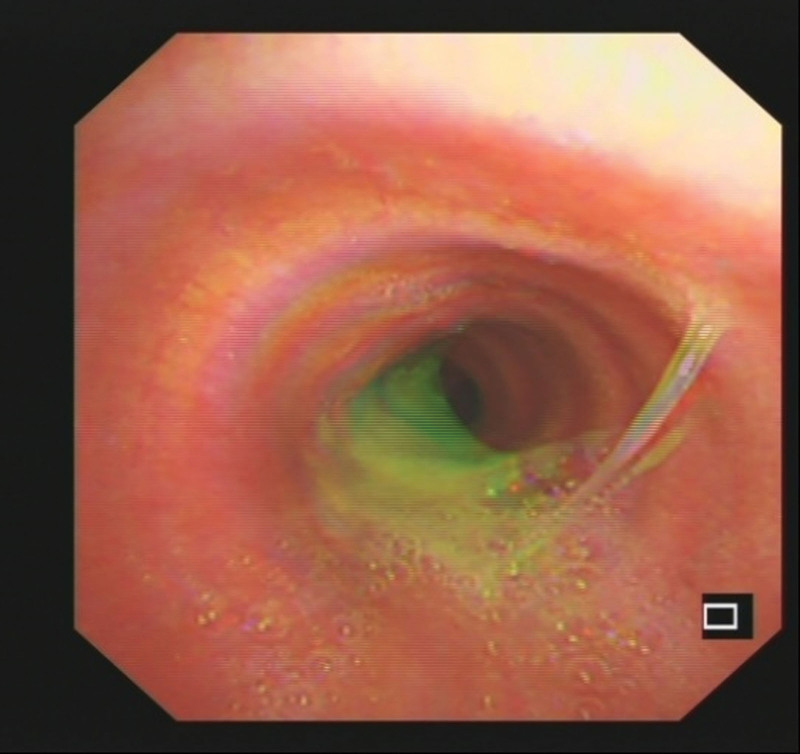
Fiberoptic bronchoscopy image of the patient revealed bile in the sputum.

**Figure 4.**
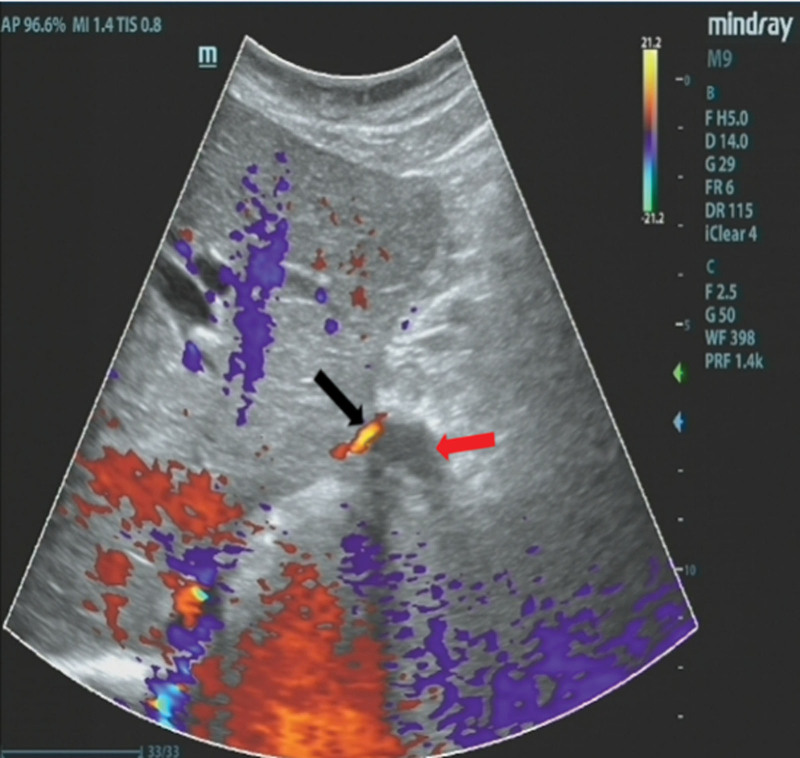
2D ultrasonography combined with Doppler ultrasonography showed blood flow (Black arrow) in front of the subdiaphragmatic fluid dark area (red arrow).

1. Laboratory workup demonstrates slightly elevated liver function tests, including total bilirubin, direct bilirubin, aspartate transaminase, alkaline phosphatase, and gamma-glutamyl transferase levels.
2. Computed tomography (CT) images showed the left intrahepatic bile duct dilatation; the left subphrenic abscess was communicated with the thoracic cavity (Fig. 1); which inferior phrenic artery was passing behind the abscess (Fig. 2).^[4]^
3. Electronic bronchoscopy showed yellow purulent fluid from the left lower lobe (Fig. 3). Bilirubin detection was positive in the sputum. According to these findings, she was diagnosed with BBF. The biliary sputum was confirmed as secondary to the BBF.^[10]^
4. 2D ultrasound combined with Doppler color ultrasound showed a dark fluid area under the diaphragm and a blood flow in front of the subdiaphragmatic fluid dark area (Fig. 4).

### 2.3. Surgical decision and minimally invasive treatment

Based on the individualized characteristics of the patient, no obvious diaphragmatic defect was found in the CT scan results, and the biliary reconstruction procedure was completed in the past months. Minimally invasive treatment was chosen for the patient.

Firstly, the percutaneous transhepatic biliary drainage (PTBD) catheter was inserted to decrease the pressure gradient between the intrahepatic duct and the abscess cavity.

Secondly, 2D ultrasound combined with Doppler color ultrasound was used to accurately locate the subphrenic abscess during the puncture (Fig. 4), an 8.5Fr tube was successfully placed in the subphrenic abscess and avoid inferior phrenic artery damage.

Thirdly, concurrent antibiotic administration and adequate nutritional support were provided to adjunctive therapies.

### 2.4. Outcome and follow-up

**Figure 5.**
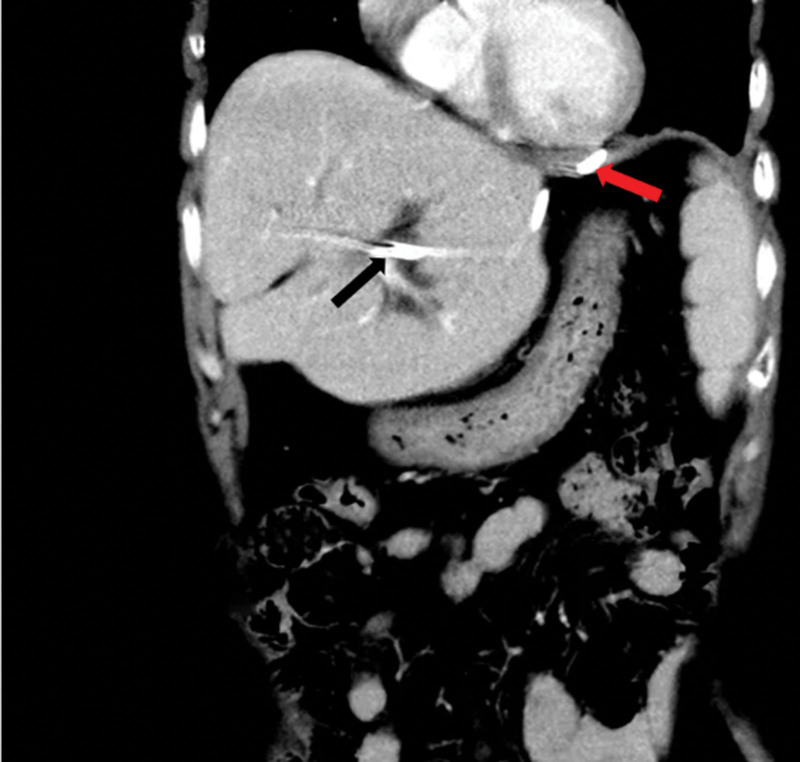
CT image scans showing 2 drainage tubes were successfully inserted into the dilated bile duct (Black arrow) and subphrenic abscess (red arrow) and adequate drainage. CT = computed tomography.

**Figure 6.**
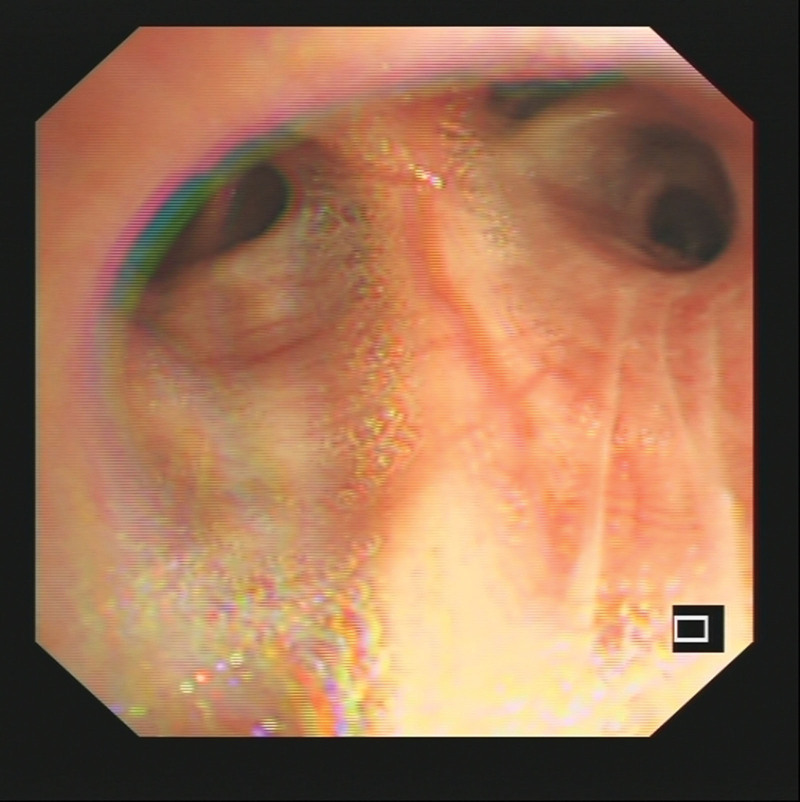
Fiberoptic bronchoscopy image of the patient revealed clean sputum.

Interestingly, the patient’s symptoms were relieved the following day, and the bilioptysis disappeared. The patient was discharged 1 week later. Two months later, CT images showed the subphrenic abscess had disappeared (Fig. 5) and no abnormalities were found during the electronic bronchoscopy reexamination (Fig. 6). The drainage tube for the subphrenic abscess and PTBD catheter were successfully removed. Follow-up for 2 years, no recurrence was found in the patient.

## 3. Discussion

BBF is an extremely rare disease that is defined as an abnormal intercommunication between the biliary tract and the bronchial tree.^[11]^ Bilioptysis (production of bilious-tinged sputum) is the pathological basis of BBF.^[10]^ Sputum analysis shows levels of direct and indirect bilirubin, which indicates the connection between the bronchi and bile ducts. Due to the multiple organ dysfunction including the thoracic and abdominal cavities, the prognosis of patients with BBF tends to a poor prognosis.^[12,13]^ Therefore, the diagnosis and treatment of BBF are significant challenges for clinicians.^[5]^

The treatment of BBF is mainly divided into systemic supportive care, minimally invasive treatment and surgical treatment. Aydin et al^[14]^ reported that they had successfully treated 3 patients with BBF by minimally invasive interventional treatments such as percutaneous drainage and endoscopic retrograde cholangiopancreatography. Compared with surgery, these techniques are less invasive, can provide good results, and have a lower risk of morbidity. However, Doyle and Sethi^[15]^ regarded that the treatment of BBF may warrant multimodal treatments, including complex surgical procedures such as explothoracotomy, omentopexy, and biliary reconstruction.

In this case, long-term indwelling PTBD catheters might have led to disruption of biliary integrity, an inflammatory reaction in the subdiaphragmatic space, the formation of subphrenic abscess’, subsequent rupture into the bronchial tree, and eventual development to BBF. In view of patient’s imaging examination, no obvious diaphragmatic defect was found in the CT scan results. Surgery was considered to be difficult due to the patient's poor general condition.

Due to the integrity of biliary destroyed, PTBD therapy was essential to reduce the biliary pressure.^[15]^ At the same time, adequate drainage of the subphrenic abscess was vital to eliminate the abnormal channel. Furthermore, intravenous antibiotics, adequate nutritional support, and drain management were important measures for the patient.^[16]^ Fortunately, the patient was successfully cured during the past 2 months.

## 4. Conclusion

In conclusion, BBF is an extremely rare disease with high mortality and a morbidity rate, and several causes can lead to this disease including congenital abnormality, hydatid illness, surgical interventions, and invasive procedures. To our knowledge, there have been no reported cases of BBF related to PTBD treatment, which makes it difficult to conduct large-scale case studies. The therapeutic procedure of BBF involves an individualized treatment plan, minimally invasive surgical techniques, and the application of a multidisciplinary collaborative approach.

## Author contributions

**Investigation:** Bo-Da Lian, Wen-Yi Zhou.

**Project administration:** Bo-Da Lian, Wen-Yi Zhou.

**Resources:** Bo-Da Lian, Jiang Peng.

**Supervision:** Bo-Da Lian, Wen-Yi Zhou, Jiang Peng.

**Validation:** Bo-Da Lian, Xin Zhang, Yong-Gang Wang.

**Writing – original draft:** Bo-Da Lian.

**Writing – review & editing:** Bo-Da Lian.

**Methodology:** Kang Zhao, Xin-Tian Wang, Zi-Li He.

**Conceptualization:** Chen Chen, Xin-Tian Wang, Zi-Li He.
